# The Role of the JAK–STAT Pathway in Childhood B-Cell Acute Lymphoblastic Leukemia

**DOI:** 10.3390/ijms25136844

**Published:** 2024-06-21

**Authors:** Karolina Joanna Ziętara, Kinga Wróblewska, Monika Zajączkowska, Joanna Taczała, Monika Lejman

**Affiliations:** 1 Student Scientific Society, Independent Laboratory of Genetic Diagnostics, Medical University of Lublin, 20-093 Lublin, Poland; kar.zietara@gmail.com (K.J.Z.); kinga.wroblewska.kw@gmail.com (K.W.); zajaczkowskam292@gmail.com (M.Z.); 2 Faculty of Medicine, Medical University of Warsaw, 02-091 Warszawa, Poland; taczalajoanna@gmail.com; 3 Independent Laboratory of Genetic Diagnostics, Medical University of Lublin, 20-059 Lublin, Poland

**Keywords:** childhood, B-cell acute lymphoblastic leukemia, genetic aberrations, JAK–STAT, treatment

## Abstract

B-cell lymphoblastic leukemia is a hematologic neoplasm that poses a serious health concern in childhood. Genetic aberrations, such as mutations in the genes *IL-7, IL7R, JAK1, JAK2, TLSP*, *CRLF2*, and *KTM2A* or gene fusions involving *BCR*::*ABL1*, *ETV6*::*RUNX1*, and *PAX5*::*JAK2*, often correlate with the onset of this disease. These aberrations can lead to malfunction of the JAK–STAT signaling pathway, which is implicated in various important biological processes, including those related to immunology. Understanding the mechanisms underlying the malfunction of the JAK–STAT pathway holds potential for research on drugs targeting its components. Available drugs that interfere with the JAK–STAT pathway include fludarabine, ruxolitinib, and fedratinib.

## 1. Introduction

Cancer is one of the major causes of death among children worldwide. According to the WHO, in 2022, leukemias accounted for 28.8% of all cancers in children under the age of 19, followed by brain tumors and non-Hodgkin’s lymphomas [1,2]. Acute lymphoblastic leukemia (ALL), which derives from precursor B (more than 80% of ALL) and T lymphocyte lineages, is the most common childhood leukemia [3,4]. The foundation for the development of this type of leukemia often involves genetic alterations including chromosomal rearrangements, hyperdiploidy, hypoploidy, sequence changes, and DNA copy number changes. Some of the most significant subtypes found in B-cell acute lymphoblastic leukemia (B-ALL) are *MLL (KMT2A)* rearrangements, *ETV6::RUNX1* translocation, *ETV6::RUNX1*-like ALL, *BCR::ABL1* Philadelphia chromosome ALL, Philadelphia chromosome-like ALL, *ZNF384*-rearranged ALL, *PAX5*-rearranged ALL and *CRLF2*-rearranged ALL [5,6,7]. Several of the genetic changes directly or indirectly affect the Janus kinase–signal transducer and transcription activator (JAK–STAT) pathway, the proper functioning of which determines the maintenance of stem cells, the process of differentiation and proliferation of immune cells, hematopoiesis, and the pro-inflammatory response. The various B-ALL genetic subtypes associated with the functioning of the JAK–STAT signaling pathway represent research potential regarding new therapeutic targets and methods in childhood leukemia [8,9]. The following article describes abnormalities in the mechanism of action of the JAK–STAT pathway implicated in selected genetic alterations associated with childhood B-ALL, alongside available and experimental therapeutic options.

## 2. Background

### 2.1. JAK–STAT Signaling Pathway Components and Their Primary Functions

The JAK–STAT signaling pathway holds significant importance in numerous biological processes within the human body, thus assuming a pivotal role in the pathogenesis of various diseases, notably including those affecting the immune system. This pathway comprises three principal components: ligand–receptor complexes, Janus kinases (JAKs), and signal transducers and activators of transcription (STATs) [10,11].

Either cytokines or growth factors as ligands can engage tyrosine kinase-associated receptors, leading to ligand–receptor complexes. The binding of a ligand to its receptor activates JAK, facilitated by ligand-mediated receptor multimerization. The proximity of JAKs enables trans-phosphorylation. The activated JAKs then phosphorylate specific tyrosine residues on the receptor’s intracellular domain. Subsequently, STAT proteins are recruited, activated, and phosphorylated on tyrosine residues by the JAKs [11,12,13]. In mammals, four kinases belong to the Jak family: Jak1, Jak2, Jak3, and Tyrosine kinase 2 (Tyk2). The genes encoding these kinases in humans are located on different chromosomes. Specifically, the *JAK1* gene is located on chromosome 1p.31.3, and *JAK2* is on 9p24.1, while *Jak3* and *TYK2* are clustered on chromosome 19p13.11 and chromosome 19p13.2, respectively. These kinases are generally ubiquitously expressed, except for JAK3, which is mainly expressed in hematopoietic cells [14,15,16,17,18,19]. JAKs consist of four domains in the following order: N-terminal FERM (band 4.1, Ezrin, Radixin, Moesin) domain, SH2 domain, pseudokinase domain (JAK Homology 2, JH2), and C-terminal kinase domain (JH1). The first two domains, FERM and Src-homology 2 (SH2), are tightly associated and form a cohesive unit linked to the peptide receptor and its Box1 and Box2 motifs. Their primary function is to bind JAKs to the cytokine receptor. The subsequent domain, the pseudokinase, exhibits catalytic inactivity, while it binds ATP and assumes a pivotal role in modulating the activity of the C-terminal kinase domain. The last domain is active and has catalytic activity, being responsible for substrate phosphorylation of the cytokine receptor, thereby activating STAT transcription factors [20,21,22,23].

The third component of the JAK/STAT pathway consists of inactive protein dimers that are located in the cytoplasm and belong to the STAT family. Seven STAT subtypes have been identified in mammals: STAT1, STAT2, STAT3, STAT4, STAT5a, STAT5b, and STAT6. Upon the initiation of signaling by various ligands, such as cytokines, growth factors, interleukins, and hormones, these proteins become activated. Their role involves transmitting signals from the ligands and promoting the transcription of specific genes. [20,24]. Each STAT is composed of distinct domains arranged in the following order: the N-terminal domain, the coiled-coil domain (CCD), the DNA binding domain (DBD), the linker region, the SH2 domain, and the C-terminal region containing the transactivation domain (TAD) [20,25,26]. The N-terminal domain is responsible for interacting with transcription factors, cofactors, receptors, and STAT dimers by binding to the DNA regions where the binding sites of STAT proteins are located [20,25]. The adjacent CCD domain contains a surface to which proteins, among other regulatory factors, attach [26]. The DBD is responsible for chromatin binding and induction of transcription, with STAT proteins acting as transcription factors [20,26]. The region connecting this domain and the SH2 domain is known as the linker region, serving as the bridge between them and participating in the transcriptional regulation of certain STAT proteins [20,25,26]. JAK phosphorylates the tyrosine residue (pY) on STAT molecules, thereby indirectly activating the SH2 domain through the recognition of phosphotyrosine motifs within cytokine receptors. The active SH2 domain serves as a dimerization site for two STAT proteins. Following homo- or heterodimerization, they are translocated to the cell nucleus, where they initiate gene transcription by binding to gamma-activated sequences (GASs). Within the TAD, serine phosphorylation occurs, facilitating interactions with cofactors and transcriptional activators, thereby enhancing STAT transcriptional activity. Moreover, the TAD is responsible for regulating protein stability by inhibiting autophosphorylation [26,27].

**Figure 1:**
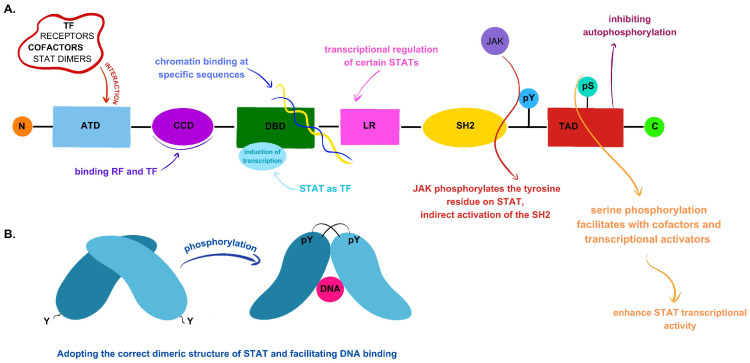
STAT domains and their functions. (**A**) From the left, the following domains of STAT are shown: the amino terminal domain (ATD), the coiled-coil domain (CCD), the DNA binding domain (DBD), the linker region (LR), the SH2 domain, and the transactivation domain (TAD) on the C-terminal end. Next to each domain, the particular functions have been presented. Abbreviations: TF—transcriptional factors, RF—regulatory factors, pY—tyrosine residue, and pS—serine residue. (**B**) The formation of the proper dimeric structure of STAT, which is capable of binding DNA, is shown graphically. The SH2 domain, tyrosine residue, and JAK proteins are involved in this process. Phosphorylation of the tyrosine residue by JAK indirectly activates the SH2 domain. A number of these actions induce dimeric changes in STAT.

Figure 1 shows all STAT domains and their functions.

### 2.2. The Regulation of the JAK–STAT Signaling Pathway

Regulation of the JAK–STAT signaling pathway relies on efficient activation following cytokine stimulation and inhibition of subsequent signaling in its absence [22].

#### 2.2.1. Signaling Activation via Cytokines

The activation of the JAK–STAT signaling pathway involves molecules such as cytokines, interferons, or growth factors interacting with JAKs. The binding of these substances induces multimerization or conformational changes within the receptor complex, leading to the activation of JAKs. These processes initiate a cascade of events where the phosphorylation of STAT proteins by active JAKs assumes a pivotal role. Subsequently, the phosphorylated STAT proteins dimerize and translocate into the nucleus [12,22].

Various cytokines activate different JAKs and STAT proteins. Table 1 illustrates the specific cytokine types interacting with distinct JAKs and STAT proteins, outlining their major functions in B-cell lineage development.

#### 2.2.2. Negative Regulation of the JAK–STAT Pathway

The negative regulation of the JAK–STAT signaling pathway involves three main groups of inhibitory factors, namely, the suppressor of cytokine signaling (SOCS), protein inhibitor of activated STAT (PIAS), and protein tyrosine phosphatase (PTP) groups [12].

The first group comprises eight proteins: cytokine-inducible SH2-containing protein (CIS), SOCS1, SOCS2, SOCS3, SOCS4, SOCS5, SOCS6, and SOCS7. These proteins negatively regulate the JAK–STAT signaling pathway through several mechanisms. Firstly, they prevent the recruitment of the SH2-STAT domain to the cytokine receptor by binding to the phosphotyrosine residue on this receptor. Additionally, they directly inhibit JAK activity by either binding to JAK itself or recruiting the SH2 domain to the receptor [37,38,39,40].

Another group, the PIAS group, consist of four proteins: PIAS1, PIAS2 (PIASx), PIAS3, and PIAS4 (PIASy), which interact with STAT dimers following JAK activation. PIAS proteins employ several specific mechanisms, including blocking STAT proteins and their DNA-binding activity, promoting SUMOylation (Small Ubiquitin-related Modifier), chelating transcription factors, and recruiting other cofactors to prevent STAT from binding to DNA [9,41,42].

The third group of negative regulators of the JAK–STAT pathway are PTP enzymes, which act by dephosphorylating tyrosine residues. Their targets for dephosphorylation may include active STAT or JAK proteins. By mediating the dephosphorylation and subsequent inactivation of JAK, these enzymes can also interact with the ligand–receptor complex [9,43].

## 3. Selected Genes Associated with Disorders of the JAK/STAT Pathway

### 3.1. IL-7 and IL-7R

The maturation of B lymphocytes is facilitated by multiple cytokines that transmit signals to their precursors through specific receptors. Errors and imbalances in signaling during early B-cell development at various stages can disrupt the sequential progression and ultimately promote the development of ALL from B precursor cells [44]. Among these cytokines, Interleukin-7 (IL-7) plays a crucial role in hematopoiesis. Its receptor comprises the γc chain, shared by all lymphopoietic cytokines, and the interleukin-7 alpha receptor subunit (IL-7Rα), which is encoded by the *IL-7R* gene located on the short arm of chromosome 5 (5p13.2) [45,46,47,48].

IL-7Rα, unlike its counterpart with which it forms a receptor complex, exhibits greater specific expression and is present, among others, on the surfaces of cells ranging from common lymphoid progenitors (CLPs) through pro-B to large pre-B cells and their T-cell counterparts. Positioned in this manner, IL-7 plays a pivotal role in the transition of pro-B cells to pre-B cells, and animal studies suggest that inactivation of one of the downstream cytokine signaling effectors may lead to the retention of B-cells in the pre-pro-B stage. Within hematopoietic cells, IL-7 primarily transmits information through three pathways: the JAK–STAT pathway, the phosphatidylinositol 3-kinase (PI3K)–Akt pathway, and the RAS–mitogen-activated protein kinase (MAPK) pathway [45,48].

The binding of IL-7 to its receptor induces heterodimerization and conformational changes in the constituent chains of IL-7R (IL-7Rα, γc). Consequently, constitutively bound tyrosine kinases JAK1 and JAK3 are activated, leading to their mutual phosphorylation. Subsequently, these activated JAK proteins catalyze the phosphorylation of a tyrosine residue within the cytoplasmic domain of IL-7Rα, creating a docking site for STAT1, STAT3, and STAT5. Among these, STAT5 plays a pivotal role in lymphopoiesis. Upon binding to IL-7Rα, STAT5 undergoes phosphorylation and homodimerization, translocating to the nucleus [45,47,49,50].

One of the consequential effects is the activation of the myeloid leukemia 1 (*Mcl1*) gene, which serves as a crucial regulator of the survival of B lymphocytes in early developmental stages, thereby inhibiting their apoptosis [51]. Furthermore, STAT5 is implicated in coordinating immunoglobulin gene rearrangements, thereby influencing Igk recombination in pro-B cells [45,51].

Therefore, IL-7’s influence through the JAK–STAT pathway on the early stages of lymphopoiesis profoundly shapes the subsequent development of B-cells. This process is further modulated by the E2A and EBF1 transcription factors [45,52].

Mutations in the IL-7 receptor gene can significantly disrupt lymphocyte development due to the pivotal roles played by IL-7. These mutations typically possess activating properties; by causing homodimerization of IL-7Rα, they augment its effects and consequently enhance JAK–STAT5 signaling. Aberrations involving the substitution of serine with cysteine at the 185th amino acid position in the extracellular domain or insertions or deletions in the transmembrane domain of IL-7R account for approximately 12% of cases of Ph-like ALL and 2–3% of B-ALL cases [47,53]. Frequently, these mutations coexist with loss-of-function mutations in the negative regulator SH2B adapter protein 3 (SH2B3), also known as lymphocyte adapter protein (LNK), or alterations in the DNA-binding protein Ikaros, also known as Ikaros family zinc finger protein 1 (IKZF2) [54,55].

According to a study by Ifat Geron et al., mutated IL-7Rα in human hematopoietic cells serves as the initiator of a pre-leukemic state, as observed in mice with transplanted cells harboring mutated IL-7Rα. This mutation contributes to the silencing of cyclin-dependent kinase inhibitor 2A (CDKN2A) and the aberrant expression of cytokine receptor-like factor 2 (CRLF2) in the development of BCP-ALL [53,56].

### 3.2. TLSP and CRLF2

Thymic stromal lymphopoietin (TSLP) is a type I cytokine belonging to the IL-2 family, similar to IL-7 in its effects and signaling mechanisms [57]. Similar to its counterpart, TSLP regulates the immune response and influences B-cell development by augmenting the proliferation of their fetal precursors. TSLP signals through cytokine receptor-like factor 2 (CRLF2), located in the pseudoautosomal region (PAR1) of the X and Y chromosomes, and the IL-7Rα subunit, which it shares with IL-7. Consequently, both cytokines activate identical effectors through a shared signaling pathway. Analogous to the IL-7 receptor, the TSLP receptor is made of components that are constitutively associated with members of the Janus kinase family: IL-7Rα with JAK1 and CRLF2 with JAK2. The binding of TSLP to both receptor subunits induces their heterodimerization, subsequently activating JAK1 and JAK2, which then phosphorylate the transcriptional regulator STAT5 [45,57,58,59,60,61,62,63,64].

Abnormalities that cause changes in signaling and significantly impact B-ALL development may affect each of the pathway components mentioned. They can be categorized into five main subgroups: gain-of-function mutations in CRLF2; mutations conferring JAK1 function (typically associated with T-ALL); mutations conferring JAK2 function; mutations activating the extracellular domains of IL-7Rα, leading to heterodimer formation with CRLF2; and mutations in the transmembrane domain of IL-7Rα, resulting in ligand-independent homodimerization [65].

**Figure 2:**
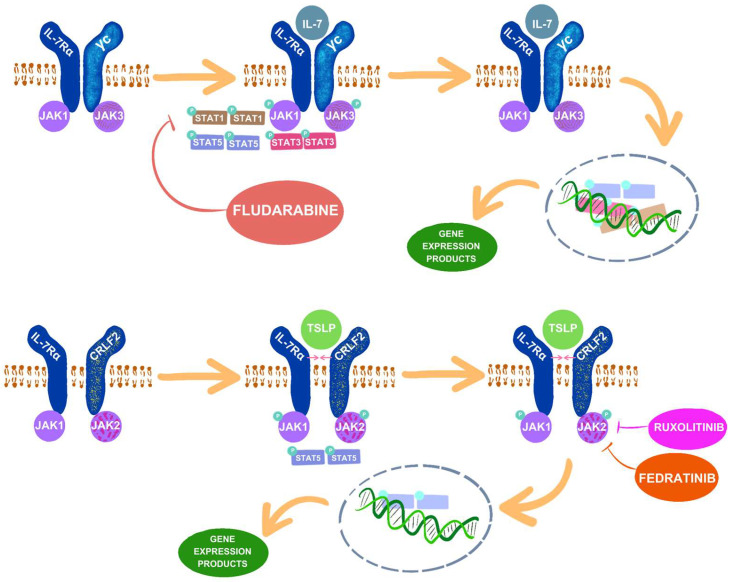
Effects of IL-7 and TLSP on the JAK–STAT signaling pathway and the action of selected inhibitors of pathway components: IL-7 leads to the activation of Janus kinases JAK1 and JAK3, as well as STAT1, STAT3, and STAT5, which are translocated to the cell nucleus, regulating gene expression. TSLP, through heterodimerization of IL-7α with CRLF2, activates JAK1 and JAK2, as well as the effector STAT5, which translocate to the cell nucleus and influences gene expression. Fludarabine is an inhibitor of STAT1, while ruxolitinib and fedratinib are JAK2 inhibitors. By preventing the activation of their target molecules, these drugs exert inhibitory effects on the JAK–STAT pathway.

Figure 2 shows the effect of IL-7 and TLSP on the activation of the JAK–STAT pathway and selected drugs that inhibit components of the pathway.

#### 3.2.1. Enhancement of CRLF2 Function

Mutations in this subgroup often coincide with JAK2 abnormalities, ultimately leading to the constitutive activation of the crucial transcriptional regulator STAT5, which exists in two related isoforms, STAT5A and STAT5B, each with distinct functions. Alternations involving CRLF2 and their impact on STAT5 can induce cytokine-independent survival and proliferation of early hematopoietic cells, potentially contributing to the development of B-cell precursor acute lymphoblastic leukemia (BCP-ALL) [45].

The most prevalent causes of CRLF2 overexpression include rearrangements involving the immunoglobulin heavy chain (*IGH*) and P2Y receptor family member 8 *(P2RY8)* genes. Other changes associated with *CRLF2* overexpression include deletions in the Ikaros gene, activating mutations in *JAK2*, point mutations such as Phe232Cys, additional copies of the X or Y chromosome or the *CRLF2* gene, and rare fusions such as *CSF2RA–CRLF2*. *CSF2RA* is located in the pseudoautosomal region of the X and Y chromosomes and encodes a protein that serves as the alpha subunit of colony-stimulating factor 2, a cytokine that controls the production, differentiation, and function of granulocytes and macrophages [66,67].

Translocation between *IGH* on chromosome 14 and the pseudoautosomal region of the sex chromosome, where the *CRLF2* gene resides, results in the formation of an *IGH–CRLF2* fusion. The *P2RY8–CRLF2* fusion arises from the deletion of the promoter region of P2RY8. Both genes are located in the same chromosomal region on the X or Y chromosome, making *CRLF2* expression reliant on the *P2RY8* promoter, which confers oncogenic potential [58,68,69]. Conversely, diminished *CRLF2* expression due to deletion of colony-stimulating factor 2 receptor alpha (CSF2RA) leads to the gene being controlled by CSF2RA, a low-affinity enhancer [66,67]. The Phe232Cys substitution mutation in the extracellular domain of CRLF2 enhances its function, leading to constitutive homodimerization of CRLF2 and activating components of the JAK–STAT pathway [61,65,70].

#### 3.2.2. Clinical Significance of CRLF2 Mutations

*CRLF2* overexpression resulting from the described genetic abnormalities is detected in 15% of children with high-risk B-ALL and 47% of pediatric patients with Ph-like B-ALL [55,71]. Therefore, high expression is considered one of the characteristic features of this subtype of ALL and, according to some researchers, has the potential to be a prognostic factor when making decisions regarding treatment selection [70,72]. There is a correlation between impaired gene expression and the ethnic origin of patients. For example, in families with Latin roots, abnormalities in the *CRLF2* gene are detected five times more often than in other patients [58]. The prognosis of B-ALL in children depends on mutations in leukemic cell genes. The overexpression of *CRLF2*, which leads to constitutive activation of JAK2–STAT5, correlates with high-risk BCP-ALL and promotes poorer recurrence-free survival rates in pediatric patients [68,73,74]. Moreover, Naglaa M. Hassan and colleagues reported that patients with high *CRLF2* expression are characterized by a greater number of blasts in peripheral blood and severe organomegaly [68,75]. However, mutations causing the activation of *CRLF2* may also be receptor-independent prognostic factors in leukemia. For example, the combination of *P2RY8–CRLF2* fusion and the associated deletion in the Ikaros gene (*IKZF1*) may increase the risk of leukemia relapse [76].

### 3.3. JAK1 and JAK2

The *JAK2* gene is located on the short arm of chromosome 9 (9p24.1) and encodes a non-receptor tyrosine kinase pivotal in cytokine signaling by binding to both type I and type II cytokine receptors. Upon ligand binding, both classes of receptors undergo dimerization, facilitating transphosphorylation and activation of JAK2. Activated JAK2, via autophosphorylation of tyrosine residues on the cytoplasmic receptor, creates anchoring sites for proteins with an SH2 domain, including STAT [17,77,78].

JAK2 activation, induced by various cytokines, is crucial for proper cell function, especially in hematopoiesis [77]. JAK2 plays a fundamental role in maintaining and regulating hematopoietic stem cell (HSC) function. It associates with the thrombopoietin receptor (TPOR), governing megakaryocyte and platelet production, as well as with EPOR, GM-CSFR, G-CSFR, IL-3R, and IL-5R, making it vital for myeloid-lineage cell differentiation. Additionally, the erythropoietin receptor (EPOR) and growth hormone receptor (GHR) are homodimeric and bind solely to JAK2 [23].

JAK2 gain-of-function mutations, commonly found in ALL, typically occur in exon 16, predominantly around amino acid R683, which is altered to glycine or serine [23,78]. These mutations within the ATP-binding site of the JAK2 pseudokinase domain destabilize the inhibitory JH2–JH1 interface, disrupting autoinhibition mediated by JH2. This exposure of an interface facilitates JAK2-related cytokine receptor dimerization and subsequent activation [77,78,79].

Notably, *JAK2* mutations alone, disrupting JH2-mediated autoinhibition, may not suffice for sustained cytokine-independent JAK2 activation [22,77,79]. The lymphoid transformation is proposed to be driven by the high association of *JAK1/2* and *CRLF2* mutations [77,80].

#### Clinical Significance of *JAK2* Mutations

All cases of B-ALL with changes in *JAK2* are accompanied by *CRLF2* overexpression, facilitating the cytokine-receptor scaffolding necessary for this signaling [81]. However, it is important to note that there are various causes of *CRLF2* overexpression, and they may not always coincide with *JAK* abnormalities. *JAK* mutations, particularly *JAK2*, are identified in around 10% of BCP-ALL cases. This percentage increases to over 20% in patients with Down syndrome who also have ALL [23,82,83]. It is noteworthy that *JAK2* mutations occur more frequently in patients diagnosed with Ph-like ALL, a high-risk subtype characterized by gene expression patterns resembling Ph+ ALL, despite the absence of the chromosomal translocation t(9;22)(q34;q11) [58,59]. Among subjects showing CRLF2 changes simultaneously with JAK2 variants, poorer treatment outcomes for ALL are observed [84,85].

### 3.4. BCR::ABL1 Fusion

The *BCR::ABL1* oncoprotein associated with Ph+ ALL directly and independently activates the JAK2–STAT pathway. The *BCR* gene, located on the long arm of chromosome 22 (22q11.2), encodes the breakpoint cluster region protein, regulating cellular signaling processes. Conversely, the *ABL1* gene, a proto-oncogene situated on the long arm of chromosome 9 (9q34.1), encodes a protein tyrosine kinase that influences cell division, adhesion, differentiation, and stress responses [86,87].

A reciprocal translocation of chromosomes 9 and 22, t(9;22)(q34;q11), results in the Philadelphia chromosome (Ph), observed in the transformed HSCs, and the formation of the *BCR::ABL* fusion gene [86,87]. Depending on the site of the break, three protein isoforms are distinguished: a variant with a molecular weight of 185–190 kDa (p190), commonly found in ALL; a variant with a mass of 210 kDa (p210), prevalent in chronic myeloid leukemia (CML); and the rarest variant, with the highest molecular weight of 230 kDa (p230) [87]. The constitutively activated *ABL1* catalytic domain, due to fusion with *BCR*, drives *BCR–ABL1* activity, enabling direct phosphorylation of cytokine receptors by the kinase protein, thereby activating the JAK2/STAT pathway [86,88,89]. Gene fusion alters JAK2–STAT pathway components’ activity by directly affecting STAT5 or mediating JAK2 phosphorylation independently. Both actions ultimately enhance signaling, promoting cell proliferation and increased survival [90,91]. Moreover, studies by Run Qina et al., using mouse models, revealed altered functions of STAT6 in the context of the described translocation. The activated cell cycle regulator enhances *c-Myc* transcription, intensifying the pathogenicity of ALL cells and worsening the prognosis. Their research also distinguished Ph+ ALL from Ph+ CML based on the subtypes of the BCR::ABL fusion protein, P190 and P210, respectively. They concluded that Ph+ ALL exhibits more pronounced activation of the JAK2–STAT6 pathway than Ph+ CML [92].

### 3.5. ETV6::RUNX Fusion

The t(12;21)(p13;q22) translocation results in the fusion of two genes: *ETV6* (also known as *TEL*) and *RUNX1* (also known as *AML1*) [93]. *ETV6* encodes an ETS family transcription factor crucial for hematopoiesis and vascular network development [94]. In contrast, *RUNX1* encodes a protein essential for normal hematopoiesis [95]. This fusion represents the most common genetic alteration in ALL and has various functional effects, including repression of *RUNX1*-dependent transcription, disruption of wild-type *ETV6* activity, induction of *EPOR* expression, and activation of STAT3 through phosphorylation [93,96,97,98,99]. It disrupts the JAK–STAT pathway by overactivation of STAT3, typically phosphorylated by JAK2 [99]. In leukemias, the visible overactivation of STAT3 accelerates leukemia cell proliferation, blocks leukemia cell differentiation, and inhibits apoptosis of leukemia cells [100,101].

As mentioned before, the fusion of these genes is often present in leukemias; however, the presence of the fusion alone does not lead to the occurrence of these diseases [102]. The genetic alternation occurring in B-ALL with this fusion is *EPOR* mutation, which has a crucial effect on the development of leukemia. This gene encodes the receptor responsible for binding erythropoietin but also for the activation of JAK2 tyrosine kinase [103,104]. Overexpression of *ETV6::RUNX* fusion and the presence of EPO simultaneously in cells result in the activation of STAT5. Additionally, this process is JAK2-dependent and cannot happen in the presence of a JAK2 inhibitor [102].

### 3.6. KMT2A

*KMT2A* (also known as *MLL*), located on human chromosome 11q23, encodes a lysine methyltransferase serving as a transcriptional coactivator essential in regulating gene expression during early organism growth, formation, and hematopoiesis [105,106]. Mutation of *KMT2A* involves translocations involving certain genes, particularly *AFF1*, *MLLT3*, and *MLLT1*, leading to the formation of oncogenic chimeric proteins [107]. *KMT2A* mutations occur in 5% of ALL cases, resulting in mixed-lineage leukemia rearrangement (MLL-r), cases of which exhibit worse outcomes than non-MLL cases [108,109]. These differences include increased white blood cell counts at onset, insensitivity to traditional chemotherapeutic agents, low complete remission rates, a relatively high incidence of central nervous system (CNS) involvement, and shortened survival. MLL-r ALL depends on JAK/STAT-mediated inflammatory signals in leukemia development [107,108,109].

### 3.7. PAX5::JAK2

*PAX5*, located on chromosome 9p13, is a transcription factor belonging to the paired box (PAX) family, critical in early organism development. Changes in its gene expression are implicated in cancer development. Specifically, this gene encodes a protein activating the B-cell lineage and is active in early B-cell development stages, not in later ones [110].

Rearrangement of *PAX5* is observed in 2.5% of B-cell precursor ALL, with particular emphasis on the *PAX5::JAK2* fusion, which plays an important role in tumorigenesis [111,112]. This fusion produces a protein that includes the *PAX5* DNA-binding region and the constitutively active JAK2 kinase domain. It disrupts the normal transcriptional program regulated by *PAX5* while also initiating the JAK–STAT signaling pathway by enhancing *STAT5* expression. This dual effect on crucial pathways suggests a potential mechanism facilitating leukemia development [113].

## 4. JAK/STAT Inhibitors as Therapeutic Targets

JAK–STAT signaling is one of the key mediators of information transfer, tasked with activating transcription proteins engaged in performing various roles, including those related to lymphopoiesis. Given the significant link between abnormal JAK–STAT signaling and B-ALL development, ongoing research is being conducted on the use of transducer components as therapeutic targets. Targeting JAK proteins, which initiate the activation of transcription regulators, holds promise for achieving improved therapeutic outcomes and potentially reducing treatment-related side effects in patients. The effect exerted by JAK inhibitors on cells is to block the active sites of kinases, which prevents proper signal transduction and abolishes the effects caused by cytokines [114].

The therapeutic potential of JAK inhibitors has been substantiated by ex vivo studies on cell lines, demonstrating that inhibiting JAK and thereby preventing STAT activation leads to restricted cell development and increased apoptosis [114,115].

JAK inhibitors can be divided based on the selectivity of action (selective or non-selective) as well as the method of binding and type of interaction with amino acids in the Janus kinase. The second classification method includes reversible inhibitors, called competitive inhibitors (divided into allosteric JAK inhibitors and ATP-competitive inhibitors) and irreversible, or covalent, inhibitors (their target is JAK3, which is associated with T-ALL and therefore will not be discussed). Notable examples of competitive inhibitors are ruxolitinib and the newer-generation drug fedratinib, both well known for their therapeutic potential in ALL [116,117].

### 4.1. Ruxolitinib

Ruxolitinib is a tyrosine kinase inhibitor (TKI) that selectively targets subtypes JAK1 and JAK2 by competing with ATP in the catalytic sites of the aforementioned kinases [117]. It is administered orally and is approved for various treatments, including myelofibrosis, polycythemia vera, and steroid-refractory graft-versus-host disease in the setting of allogeneic stem-cell transplantation [118]. Ruxolitinib acts by attenuation of the inflammatory state caused by constitutive JAK/STAT activation, and it also induces nonspecific myelosuppression [119]. Its role as a TKI has shown potential as a treatment option for various types of leukemias characterized by JAK–STAT pathway aberrations [120].

A study conducted by Downes et al. presents results from the treatment of 12 patients with Ph-like ALL with ruxolitinib. In all the patients, there was a gene mutation leading to activation of the JAK–STAT pathway (e.g., gene fusion affecting *JAK2*, overexpression of *CRLF1*, etc.). The majority of them (*n* = 9) achieved completed or partial remission [121].

Ruxolitinib has also been used in treatment of MLL-r, specifically in cases with mutation of the *KMT2A* gene [109]. This type of leukemia is associated with poor prognosis, and few treatment options are available [109]. Laboratory data confirm the positive effects of the use of ruxolitinib in MLL-r leukemia, but further clinical research must be provided before it is used generally in clinical practice [109].

Despite promising results from numerous studies, in vitro models suggest that administering ruxolitinib as monotherapy may lead to the development of mutations conferring resistance to the drug or cross-resistance to multiple JAK-inhibitor therapies. Data from murine models suggested that cells acquired JAK2 kinase domain mutations that were resistant to multiple type I JAK inhibitors after targeted monotherapy. Therefore, ruxolitinib should be considered only as an adjunct to standard ALL treatment [121]. Nevertheless, the use of ruxolitinib as an additional drug may result in an enhanced effect in the treatment of Ph-like ALL because of its synergistic interaction with certain anti-leukemic drugs. A study performed by Böhm et al. in 2021 [85] revealed that the best effect was attainable with glucocorticoids, topoisomerase I and II inhibitors, microtubule-targeting agents, and antimetabolites.

### 4.2. Fedratinib

Fedratinib, an oral selective JAK2 inhibitor targeting FMS-like tyrosine kinase-3 (FLT-3), was approved by the Food and Drug Administration (FDA) in 2019 for the treatment of primary or secondary myelofibrosis, eight years after the introduction of ruxolitinib [116,122]. The use of fedratinib, this time in B-ALL, was tested in combination with venetoclax in cell lines that were then injected into mice. Venetoclax is a selective B-cell lymphoma 2 (Bcl-2) protein inhibitor with anti-apoptotic properties, which play an important role in high-risk B-ALL, especially with *KMT2A* mutations [123]. The combination of these two drugs has made it possible to achieve better effects, with the exception of cell lines that do not express the Flt3 tyrosine kinase, responsible for promoting growth and inhibiting apoptosis in the early stages of hematopoiesis. The conducted research proved the validity of combining the two inhibitors in order to achieve enhanced anti-leukemic effects and showed the correlation between the effect of the drugs and Flt3 overexpression, which often occurs in B-ALL with poor prognosis [123,124]. Given that the phosphorylation of STATs and their movement from the cytosol to the nucleus triggers the transcriptional activation of specific genes, there has been interest in inhibiting this mechanism. For this reason, research has begun on STAT inhibitors that may target the SH2 domain, mRNA-binding domain or DNA-binding domain. Currently, the only FDA-approved agent for the leukemia treatment is a STAT1 inhibitor, a purine analogue called fludarabine [114,125]. This drug is administered intravenously in the form of a monophosphate prodrug (F-ara-AMP), which is quickly converted into an intracellularly distributed metabolite: F-ara-A. In the next step, intracellular phosphorylation occurs, leading to the formation of the active metabolite fludarabine triphosphate, which is incorporated into DNA and RNA, thus inhibiting the synthesis of these nucleic acids [126]. The precise mechanism and effect of fludarabine on STAT1 are not fully elucidated; however, it has been shown to reduce STAT1 phosphorylation, thereby inhibiting its activity [127,128].

Additionally, fludarabine was combined with cyclophosphamide in a lymphodepletion regimen prior to chimeric CD19 antigen receptor (CAR) T-cell therapy. Research has demonstrated that the combination of these drugs resulted in significant improvements in outcomes in patients with relapsed or refractory B-ALL [128,129,130].

## 5. Conclusions

Over the years, there has been notable progress in the development of both diagnostic modalities and therapeutic protocols for pediatric B-ALL, resulting in considerable achievements. This advancement has manifested in an impressive overall survival rate of 90% among affected children. However, despite these strides, substantial challenges persist within this domain, and the trajectory of medical innovation remains uninterrupted. This is exemplified by ongoing endeavors to conceptualize novel therapeutic interventions targeting the JAK–STAT signaling pathway, which is unequivocally implicated in the pathogenesis of B-ALL.

Independent mutations affecting constituents of this pathway, alongside those implicating its associated genes, engender dysregulation of gene expression and cellular processes. Such perturbations contribute significantly to the initiation, progression, and prognostication of leukemia. Consequently, JAK–STAT transducers and effectors, ubiquitous across multiple signaling cascades, emerge as auspicious targets for inhibitors such as ruxolitinib, fedratinib, and fludarabine. These agents hold considerable promise for incorporation into the therapeutic arsenal against B-ALL characterized by the aforementioned genetic alterations. Continued research and clinical trials will be crucial in harnessing their full potential and improving outcomes for pediatric patients affected by this challenging disease.

## Figures and Tables

**Table 1:** Janus kinases and signal transducer and transcription activators activated by selected cytokines and their meaning in B-cell lineage development.

Cytokine	JAKs	STATs	Functions	References
IL-7	JAK1, JAK3	STAT1, STAT3, STAT5a, STAT5b	B-cell progenitor growth, differentiation, survival, and proliferation	[9,15,20,28,29,30,31,32,33,34,35,36]
IL-9	JAK1, JAK3	STAT1, STAT3, STAT5a, STAT5b	Development and stimulation of B-cells	
IL-21	JAK1, JAK3	STAT3, STAT5a, STAT5b	Maturation and development of B-cells; production of memory B-cells from naive precursors	
IL-5	JAK1, JAK2	STAT1, STAT3, STAT5a, STAT5b	B-cell growth, differentiation, and survival	
TLSP	JAK1, JAK2	STAT1, STAT3, STAT5a, STAT5b	B-cell proliferation and stimulation	
